# A Pilot Study of Ketamine versus Midazolam/Fentanyl Sedation in Children Undergoing GI Endoscopy

**DOI:** 10.1155/2011/623710

**Published:** 2011-05-16

**Authors:** Jenifer R. Lightdale, Paul D. Mitchell, Meghan E. Fredette, Lisa B. Mahoney, Steven E. Zgleszewski, Lisa Scharff, Victor L. Fox

**Affiliations:** ^1^Division of Gastroenterology, Children's Hospital Boston, 300 Longwood Avenue, Boston, MA 02115, USA; ^2^Children's Hospital Boston, Boston, MA 02115, USA; ^3^University of Connecticut School of Medicine, Farmington, CT 06030, USA; ^4^Boston University School of Medicine, Boston, MA 02118, USA

## Abstract

*Background*. Ketamine sedation has been found superior by physician report to traditional sedation regimens for pediatric endoscopy. *Goal*. To objectively compare sedation with ketamine versus midazolam/fentanyl for children undergoing gastrointestinal endoscopy. *Study*. Patients received one of two regimens and were independently monitored using a standardized rating scale. *Results*. There were 2 episodes of laryngospasm during ketamine sedation. Univariate analyses showed patients sedated with ketamine (*n* = 17) moved more (median 25% of procedure time versus 8%, *P* = .03) and required similar low levels of restraint (0.83% versus 0.25%, *P* = .4) as patients sedated with midazolam/fentanyl (*n* = 20). Age-adjusted analyses suggested that patients sedated with ketamine were comparably more quiet (*P* = .002). *Conclusions*. A pilot trial of ketamine at our institution was associated with episodes of laryngospasm. In addition, children sedated with ketamine moved and required restraint similarly to patients sedated with midazolam/fentanyl. Physician perceptions may be affected by the fact that children who received ketamine were less likely to vocalize distress.

## 1. Introduction

Moderate sedation is defined as a level of consciousness at which pain is successfully diminished, while the abilities to maintain a patent airway and to respond to light stimuli are maintained [1]. While many pediatric endoscopists report performing all of their procedures with general anesthesia, approximately a third continue to report administering moderate sedation [2] using a variety of endoscopist-administered sedation regimens [3, 4]. Many institutions use a sedation regimen that combines a narcotic with a benzodiazepine. More recently, ketamine has been suggested as a preferred regimen—both in terms of its safety and effectiveness [5–8]. 

Comparisons of sedation regimens for endoscopy are fundamentally dependent upon clear outcome measures. Whereas safety of sedation can be quantitatively determined by measuring adverse events, assessing the effectiveness of sedation has been less clearly defined. Historically, effectiveness of sedation for children undergoing endoscopy has been described either in terms of successful procedure completion, or has been based upon subjective staff ratings of intraprocedural sedation adequacy [4, 7]. Neither of these measures fully account for immobilization and patient cooperation as ideal outcomes of sedation [8]. 

Indeed, while many children tolerate moderate sedation and undergo GI procedures safely, some can become quite agitated despite adequate dosing of sedatives. Children who are not effectively sedated may struggle, vocalize distress, and require manual restraint [5]. The risks of GI procedures in uncooperative or moving patients are considerable and include perforation of the intestinal tract and hemorrhage [9, 10]. While restraint can be used to keep patients safe, it is considered psychologically harmful to children [11]. 

Prior studies of ketamine sedation for pediatric gastrointestinal endoscopy have been retrospective in nature, and have used chart review to identify any clinical concerns for inadequate sedation [7, 8]. The aim of our prospective study was to pilot the use of ketamine at our institution using independent monitors and a standardized behavioral rating scale to formally compare it with our institutional standard sedation regimen of midazolam + fentanyl. Although human subject protection concerns precluded randomization of patients to sedation arms, we hypothesized that selection biases inherent in this quality research study would favor the ketamine arm, as physicians would specifically have requested ketamine for those patients they felt would be better sedated with it. Accepting that bias as unavoidable, we sought to confirm the hypothesis that patients receiving ketamine would move less, vocalize distress less, and require less restraint by clinical staff during GI procedures than patients receiving standard sedation with midazolam and fentanyl. A secondary aim was to compare the safety of these two common regimens.

## 2. Methods

With approval from our hospital's Sedation Committee to pilot the use of ketamine in our unit in approximately 15–20 patients, we prospectively studied a convenience cohort of children referred for endoscopy at Children's Hospital Boston between March 2006 and July 2007 with Institutional Review Board approval. Patients were evaluated and a sedation regimen was selected based on the clinical judgement of their gastroenterologists at the time of procedural scheduling. Appropriate informed consents for the procedure and the study were obtained on the day of endoscopy. 

Per institutional protocol, all patients were sedated to achieve a goal depth of moderate sedation (Ramsay Level 4). Per study protocol, all patients who received ketamine were administered an initial 1 mg/kg bolus dose of ketamine (max dose 70 mg), followed by a maximum of 2 additional bolus doses administered as necessary every 5 minutes. The comparison group was comprised of a convenience sample of patients who received our unit's more standard moderate sedation regimen of midazolam (0.05–0.3 mg/kg IV, max dose 15 mg, every 3 minutes) and fentanyl (1–5 *μ*/kg, max dose 250 *μ*, every 5 minutes). 

In both study groups, sedation was actively administered by credentialed gastroenterologists. Midazolam/fentanyl was administered or supervised by one of five different attending gastroenterologists who performed such cases during the study period. All ketamine sedation cases were performed by a single endoscopist (VF), with observation by a staff anaesthesiologist (SZ) who was also in the procedure room. Procedures in both arms were also attended by two nurses educated in sedation pharmacology. In addition, independent monitors measured patient movements, need for restraint and vocalization of distress using the Ohio State University Behavioral Rating Scale (OSUBRS) [12]. 

The OSUBRS is a unique monitoring tool of sedation tolerability that has been shown to have excellent reliability and validity in children undergoing gastrointestinal procedures [13]. The distinct advantage of the OSUBRS over other available behavioral scales used in monitoring pediatric sedation is that it allows for continuous measurement of the tolerability of sedation throughout the duration of a procedure [12]. The OSUBRS uses a computer program to capture mutually exclusive classifications of behavior (e.g., crying versus being quiet) that are recorded continuously over time. Medical training is not required to use the rating scale, nor is knowledge of sedation or procedural technicalities [14–16].

**Figure 1:**
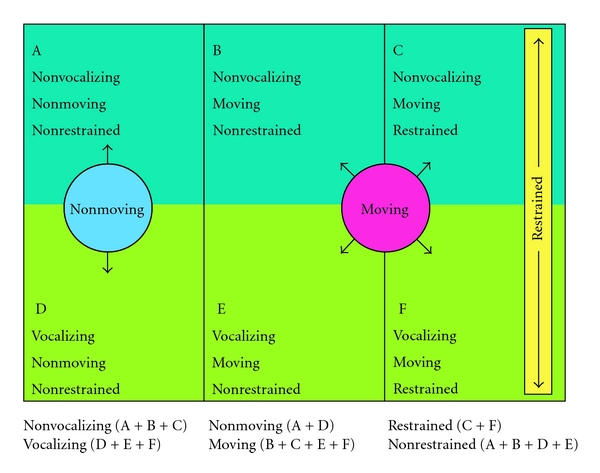
Explanation of variables in the Ohio State University Behavioral Rating Scale (OSUBRS).

For this study, an independent monitor trained to use the OSUBRS rating scale was present during each procedure. The monitor sat at a computer and pressed specific letters on a keyboard (e.g., “A” for quiet, nonmoving, nonrestrained, “F” for vocalizing distress, moving, restrained) to record the behavioral states over the course of the procedure (Figure 1). An internal clock recorded the length of each behavioral state as well as the overall length of the procedure. Following each procedure, a study coordinator recorded the total sedative dosage, depth of sedation, duration of sedation, duration of the procedure, and any observed intraprocedural adverse events from the medical record. In addition, the study coordinator reviewed charts for clinical documentation suggesting inadequate sedation, as consistent with methodology used in prior literature.

### 2.1. Statistical Analysis

We performed an intention-to-treat analysis. OSUBRS outcomes were calculated as the percentage of overall procedure time a patient was observed vocalizing, moving, or requiring restraint (Figure 1). There are 6 unique combinations of states: 3 classifications of movement (no movement, unrestrained movement, retrained movement) × 2 classifications of vocalization (yes/no). Due to the skewed nature of the outcomes, continuous data are presented as median (IQR) and two-group comparisons use the Wilcoxon rank-sum test. Categorical variables are presented as *N* (%), and group comparisons are tested with the Pearson *χ* ^2^ statistic if all expected cell-counts are ≥5, and Fisher's exact test otherwise.

Analysis of covariance was used to determine covariates independently associated with each outcome. Covariates under consideration included type of sedation, age, gender, American Society of Anesthesiology (ASA) class, and weight. Age and weight were highly correlated, and therefore not included in any model simultaneously. Since gender was not statistically significant in any model and weight had a weaker effect than age, all models include only type of sedation and age at the time of the procedure. An interaction between type of sedation and age was modeled to determine if there were differential effects for sedation type by age. For outcomes that were skewed, the Blom normal score from the ranked data, **y**_*i*_ = ^Φ − 1^(**r**_*i*_ − 3/8)/(**n** + 1/4), was used as a normalizing function. Diagnostics to check the fit of the model included a histogram of the residuals and plots of the residuals against the predicted values and each covariate included in the model. The results for the rank-transformed and nonranked outcomes were similar, and therefore only the latter are reported. 

All tests were two-sided and conducted at the 0.05 level of significance. Data analyses for this paper were generated using SAS/STAT software, Version 9.1 of the SAS System for Windows, SAS Institute Inc., Cary, NC, and SPSS for Windows, Release 14.0, 2005, Chicago, IL.

## 3. Results

A total of 17 children were selected by their physicians to receive ketamine sedation during the pilot study period. Their data was compared with a cohort of 20 children who received midazolam/fentanyl. Patient characteristics reflected the selection bias inherent in the study and showed the two study groups were not similar with respect to patient characteristics (Table 1). Median age and weight of children who received ketamine sedation was less than half that of children in the midazolam/fentanyl group (*P* = .004 and *P* = .005, resp.). Furthermore, the proportion of male gender in the ketamine group (76%) was higher than in the midazolam/fentanyl group (45%; *P* = .052). Similarly, the rate of colonoscopy in the ketamine group (47%) was more than double that of the midazolam/fentanyl group (20%; *P* = .003). ASA physical status classification scores were similar across patient groups, with 86% of patients documented as class I (normal healthy patient) and the remaining 14% documented as class II (mild systemic disease). 

Medical histories were similar across patient groups (data not shown). The most common conditions noted were abdominal pain (38%), followed by respiratory ailments (24%; 7/9 with asthma), allergies (22%), head/ears/eyes/nose/throat ailments (22%), and gastroesophageal reflux disease (22%). Thirty percent of children were taking medication for reflux, and 16% were taking laxatives regularly at the time of the sedated procedure. 

Esophagogastroduodenoscopy (EGD) accounted for more than half (68%) of all gastrointestinal procedures performed in this study; all other procedures were either colonoscopy (19%) or combined EGD/colonoscopy (14%). There was no statistical difference in procedure times between the two study groups. Mean sedation time was 34 ± 16 minutes for procedures that took 19 ± 14 minutes on average to perform. 

### 3.1. Safety

Two patients receiving ketamine sedation for upper endoscopy were documented to experience laryngospasm during their procedures. The first case was a 3-year-old male with abdominal pain and a history of anxiety, undergoing an upper endoscopy for a question of Celiac disease. Two 1 mg/kg boluses of ketamine were administered with a 5 minute interval between doses. The endoscope was introduced, and 4 minutes later the patient experienced laryngospasm, as detected by capnography, as well as oxygen desaturation to less than 90% SpO2. In response, an oral airway was placed, positive pressure ventilation was performed and the procedure was aborted. The second case of laryngopasm involved a 7-year-old female with complaints of dysphagia and a distant history of mild asthma who was scheduled to undergo upper endoscopy. A 30 mg/kg bolus of ketamine was administered to good sedation effect. The procedure was started and within 3 minutes halted due to laryngospasm and oxygen desaturation to a SpO2 of 50%. Positive pressure ventilation with was administered with return to full spontaneous ventilation and a normal oxygen saturation of 100%. The procedure was subsequently restarted and completed successfully.

### 3.2. Sedation Effectiveness

No patient charts reviewed in the study were noted to have documentation suggesting inadequate sedation to perform endoscopy, and all were documented to achieve institutional goal depth of sedation. In terms of prospective measurements, unadjusted analysis showed neither sedation type was strongly associated with vocalization or need for restraint (Table 2). Vocalization accounted for only a median 2.5% of procedural time (IQR 0.3–9.7%), while physical restraint was required just 0.5% of procedural time (IQR 0.0–6.7%). Patients sedated with ketamine did however move their arms, bodies, and legs more than those sedated with midazolam/fentanyl (median 25% of procedure time versus 8% of procedure time, *P* = .03).

**Figure 2:**
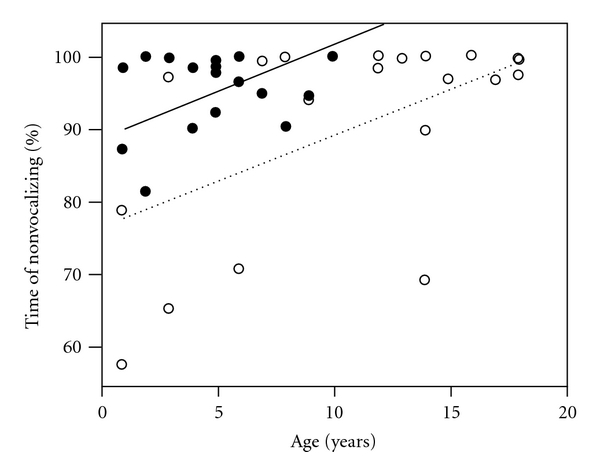
Analysis of covariance of percent time nonvocalizing on years of age, adjusted for sedation type. Ketamine sedation is represented by closed circles (●) and a solid regression line (—) while medazolam/fentanyl is represented by open circles (∘) and a dotted regression line (…). Ketamine sedation is associated with a greater percentage of time nonvocalizing compared with medazolam/fentanyl (*P* = .002), independent of the effect of age. The interaction of sedation type with age was not statistically significant (*P* = .31).

After adjusting for age, there was no difference between the two sedation types with respect to patient movement, with older patients tending to move less than younger patients, independent of sedation type (*P* = .02). Increased age was also associated with more time nonvocalizing regardless of sedation type (*P* = .001). In an age-adjusted analysis (see Figure 2), ketamine sedation was associated with a greater percentage of time nonvocalizing across all ages compared with midazolam/fentanyl (*P* = .002).

## 4. Discussion

The primary aim of our pilot study was to formally and prospectively compare the effectiveness of moderate sedation with ketamine for gastrointestinal endoscopy in children with that of more commonly used regimens combining benzodiazepines and opioids. To a great extent, this study was undertaken with the expectation that piloting ketamine in our endoscopy unit with an eye to quality and safety would reveal ketamine to be superior to our current standard of midazolam and fentanyl. We accepted a patient-selection bias, and indeed our results show that independent of age, pediatric patients who received ketamine were less likely to vocalize distress than those administered midazolam/fentanyl. On the other hand, we also found that ketamine was associated with increased (not decreased) body movement and the same need for restraint by clinical staff as children receiving midazolam/fentanyl. Such results are useful for clarifying a discussion of the comparative effectiveness of these two sedation regimens for endoscopy. 

Patients in our study were administered a particular sedative based on physician judgement. In general, we found that physicians were more likely to refer younger children for ketamine, perhaps based on a perception that midazolam/fentanyl does not adequately provide sedation in this age group and that ketamine would be more effective. We believe such selection biases should have favored the effects of ketamine. Instead, we found that patients who received ketamine required restraint for equal percentages of procedure time as their peers who received midazolam/fentanyl. 

A secondary aim of our study was to examine sedation safety, at least in terms of intraprocedural events. In our small study, there were two patients who experienced laryngospasm in the ketamine group versus no patients with adverse events in the midazolam/fentanyl group. Although ketamine has been deemed safe based on its cardiovascular and respiratory protective effect [8, 17–19], it is also associated with increased risk of laryngospasm [7, 20, 21]. Both episodes of laryngospasm in our study occurred during upper endoscopic procedures, where stimulation of the posterior pharynx may increase risk of airway complications. 

Our pilot work objectively assesses and compares effectiveness of sedation regimens for children undergoing endoscopic procedures using independent monitoring and a standardized, continuous, behavioral rating scale. Previous reports in dental and oncology populations have used global ratings by clinicians to state that ketamine reduces behavioral distress [22], increases patient cooperation [23], and reduces crying and body movements [24, 25]. In contrast, the independently obtained observations in our study are consistent with the fact that ketamine sedation increases muscular hypertonicity and stereotypical “random movements” in children [26], leading to use of restraint. The discrepancies between our investigation and prior reports are most likely due to a previous lack of standardized and objective behavioral scoring methods in head-to-head comparisons of the effectiveness of sedation regimens for gastrointestinal endoscopy. We believe the use of continuous measures over the duration of a procedure may ultimately allow a clearer definition of effectiveness as a descriptor of sedation regimens. 

The Ohio State University Behavioral Scale (OSUBS) used in this study has been previously shown to be a reliable method for evaluating behaviour in sedated children undergoing dental procedures [16, 27], and has been validated by our group in children undergoing gastrointestinal procedures [13]. In turn, effectiveness of sedation can be operationalized according to the OSUBRS. In particular, it may be plausible to define optimally effective sedation as rendering a patient “quiet, still, and unrestrained”; while a patient who is “vocalizing distress, moving, and restrained” is least effectively sedated. The use of the OSUBRS also allows measurement of the tendency for patients to move back and forth between behavioral states, as the effects of the medications, their underlying anxiety, and the stimulus of the procedure vary over time. 

There are a number of limitations to the present study that should be considered, in addition to the most important concern that the study was not randomized. In particular, the pilot nature of this study precluded adequate sample size calculations, the sample sizes analyzed were small, and study groups were unequal with regards to patient age and procedure type. Patients receiving ketamine were overall younger (median age of 5 years) and more likely to be undergoing colonoscopy compared to the fentanyl/midazolam group (median age of 12.5 years). The small sample size also precludes a true discussion of safety. Our pilot data may be useful for planning larger studies required to determine whether laryngospasm or other adverse events occurs more frequently with ketamine than has been reported previously.

Another possible limitation of this study was that it used relatively broad-based definitions of “moving” and “needing restraint”. Moving in the present study included both intentional (combative) and unintentional (drug-induced) movement. Nonetheless, regardless of reason for restraint or forcefulness of restraint, the number of staff needed for the procedure to be safely conducted remained unchanged. Ketamine, therefore, may not afford advantages for pediatric endoscopy in terms of reducing staff or resources. 

A final aspect not considered here, but worthy of investigation are more long-term adverse events associated with ketamine, as a dissociative and hallucinogenic agent. Ketamine has been observed to induce a “locked-in” state, which may prevent patients from vocalizing distress, but allow them to witness their procedure. Future studies might consider sending patients home with a diary to document their experiences after their procedure under ketamine. 

In conclusion, fentanyl/midazolam is a standard sedative regimen commonly used for GI procedures that is not totally reliable at rendering patients quiet and nonmoving. More recently, a number of studies have suggested that ketamine may provide superior and equally safe pediatric sedation [8, 19, 26, 28, 29]. Although patients in our prospective study were less likely to vocalize distress, they were still likely to move and require restraint during procedures. In addition, our study found ketamine sedation to be more associated with laryngospasm during pediatric endoscopy, which has currently limited our interest in using it further in our unit. Our study demonstrates that effective sedation relies on numerous factors and may not be adequately measured by global postprocedural ratings. With the use of objective and continuous behavioral measurements, key differences in the effectiveness of different sedation regimens for gastrointestinal endoscopy will become clearer and guidelines for improvements in pediatric sedation more likely.

## Figures and Tables

**Table 1:** Results: Patient demographics by sedation regimen.

Paient descriptives	Ketamine	M/F
	(*N* = 17)	(*N* = 20)
Age (years), *median* (IQR)	5.0 (2.5, 6.5)	12.5 (6.3, 15.8)
Gender male *n *(%)	13 (77)	9 (45)
Race (white, non-Hispanic) *n* (%)	11 (65)	17 (85)
Type of procedure *n* (%)		
EGD	9 (53)	16 (80)
Colonoscopy	7 (41)	0 (0)
EGD/colonoscopy	1 (6)	4 (20)
Weight (kg), *median* (IQR)	19 (13.7, 23.2)	44 (22.4, 61.4)
ASA level* n* (%)		
I (healthy)	14 (82)	18 (90)
II (mild systemic disease)	3 (18)	2 (10)

**Table 2:** Median percent time spent by patients receiving either midazolam/fentanyl or ketamine sedation in each behavioral state (*N* = 37).

OSUBRS measure	Behavioral state	Midazolam/fentanyl	Ketamine	P*
		Median, (IQR)	Median, (IQR)	
		Range	Range	
A	Nonvocalizing, nonmoving, nonrestrained	90.1 (67.7, 97.8) 17.8–99.9	74.5 (62.8, 85.8) 46.2–99.4	.067
B	Nonvocalizing, moving, nonrestrained	4.5 (1.7, 8.4) 0–30.9	11.7 (5.6, 21.7) 0–40.4	.023
C	Nonvocalizing, moving, restrained	0.2 (0, 1.6) 0–30.1	0.8 (0, 8.5) 0–46.4	.302
D	Vocalizing, nonmoving, nonrestrained	0.6 (0.1, 3.4) 0–41.0	0 (0, 2.6) 0–7.6	.180
E	Vocalizing, moving, nonrestrained	0.7 (0, 2.8) 0–13.7	1.1 (0, 3.8) 0–11.2	.609
F	Vocalizing, moving, restrained	0 (0, 0.6) 0–34.2	0 (0, 1.0) 0–6.4	.893

*Wilcoxon rank-sum test.
